# Therapeutic potential of miRNA-26a-encapsulated nanoparticles against hepatocellular carcinoma in a murine model

**DOI:** 10.1016/j.livres.2025.10.002

**Published:** 2025-10-22

**Authors:** Marwa Hassan, Inés Fernández-Piñeiro, Iker Badiola, Mohamed Elzallat, Alejandro Sánchez, Tarek Aboushousha, Ehab Hafiz, Eman El-Ahwany

**Affiliations:** a Immunology Department, Theodor Bilharz Research Institute, Giza, Egypt; b Department of Pharmacy and Pharmaceutical Technology, Faculty of Pharmacy, University of Santiago de Compostela, Santiago de Compostela, Spain; c Department of Cell Biology and Histology, Faculty of Medicine and Nursery, University of Basque Country, UPV/EHU, Leioa, Spain; d Nanokide Therapeutics SL, Zitek Ed, Rectorado Bajo, B° Sarriena sn, Leioa, Spain; e Genetics and Biology of the Development of Kidney Diseases Unit, Sanitary Research Institute (IDIS) of the University Hospital Complex of Santiago de Compostela, Santiago de Compostela, Spain; f Pathology Department, Theodor Bilharz Research Institute, Giza, Egypt; g Electron Microscopy Department, Theodor Bilharz Research Institute, Giza, Egypt

**Keywords:** Hepatocellular carcinoma (HCC), microRNA-26a, Nanoparticles, Diethylnitrosamine, Animal model

## Abstract

**Background and aims:**

Hepatocellular carcinoma (HCC) treatment options are limited due to the lack of effective curative therapies, and conventional chemotherapy has often demonstrated limited effectiveness and may be associated with significant toxicity. Therefore, innovative therapeutics for HCC are urgently needed. Here, we aimed to evaluate the effectiveness of microRNA (miRNA)-26a-encapsulated nanoparticles in HCC treatment.

**Methods:**

Span-Oleylamine-chondroitin sulfate were prepared and eighty male BALB/c mice were divided into four groups: (i) negative control group (saline injection), (ii) HCC group (intraperitoneal injection of diethylnitrosamine (DEN), at 50 mg/kg/week for 18 weeks), (iii) miRNA-26a-treated HCC group (intrahepatical injection of 100 nmol free miRNA-26a once weekly for four weeks), and (iv) miRNA-26a-loaded nanoparticles-treated HCC group (intrahepatic injection of miRNA-26a-loaded nanoparticles once weekly for 4 weeks, starting from Week 14 of DEN induction). Then, all mice were subjected to biochemical, genetic, histopathological, and immunohistochemical examinations.

**Results:**

The HCC group showed elevated serum levels of alpha-fetoprotein (AFP), des-gamma-carboxy prothrombin (DCP), vascular endothelial growth factor (VEGF), and tumor necrosis factor-alpha (TNF-alpha), along with upregulated hepatic expression of *P70S6K*, *transforming growth factor-beta*, *DNA methyltransferase 3 beta* (*DNMT3b*), and *Caspase-3* genes, as well as HepPar 1, Ki67, cyclin D1, and arginase 1 proteins (all *P* < 0.001). miRNA-26a treatment attenuated these changes; moreover, the miRNA-26a-encapsulated nanoparticles caused a more dramatic decrease of these markers, resulting in almost complete restoration of the normal hepatic architecture.

**Conclusions:**

Administration of miRNA-26a-encapsulated nanoparticles induced nearly total regression of HCC, suppression of cancer cell growth and angiogenesis, and induction of tumor necrosis. This study demonstrates the therapeutic efficacy of restoring the imbalanced expression of miRNA in the liver. Therefore, the clinical translation of this miRNA-based strategy warrants further investigation.

## 1 Introduction

Hepatocellular carcinoma (HCC) is a highly prevalent and fatal malignancy and ranks as the third leading cause of cancer-related mortalities globally. It accounts for around 905,677 new cases and 830,180 deaths annually and is characterized by poor clinical outcomes due to late detection, treatment resistance,^1^ and high recurrence rates. HCC underscores the urgent need for innovative diagnostics and therapeutic approaches.^2^, ^3^, ^4^, ^5^, ^6^

In early-stage HCC, there are treatment options that have the potential to cure the disease. These include surgical removal, ablation, and liver transplantation. Nevertheless, only approximately 30%–40% of cirrhotic patients who participate in surveillance programs are amenable to these sorts of therapeutic interventions.^7^ In the advanced stages of HCC, the therapeutic options are further constrained by the absence of curative treatments. Although conventional chemotherapy has shown some effectiveness, it remains only moderately effective and can induce severe side effects.^8^ Sorafenib was the first approved initial systemic treatment for advanced HCC, although studies have shown that the average survival duration is barely three months.^9^ Regorafenib is administered as a second-line medication for liver cancer patients who are experiencing progression while on treatment with sorafenib. However, the median survival period for these patients is limited to only 10.6 months.^10^ Although these therapies can improve the overall survival of HCC patients, their benefits are short-lived. Furthermore, concerns regarding resistance to these medications and their adverse pharmacological effects are growing.^11^ As a result, innovative therapeutics for HCC are urgently needed.

Specific microRNAs (miRNAs) are implicated in various biological activities, including development, cellular proliferation, apoptosis, and tumorigenesis.^12^ The primary regulatory mechanisms of miRNAs in HCC encompass processes such as cell proliferation, programmed cell death, epithelial-mesenchymal transition (EMT), angiogenesis, invasion, metastasis, and drug resistance.^13^ The discovery that individual miRNAs have the ability to target hundreds of genes and that a substantial proportion of messenger RNA (mRNA) can be controlled by miRNAs emphasizes the increasing significance of miRNA-mediated regulation.^14^ However, miRNAs’ therapeutic efficacy is restricted by their poor targeting ability, short circulation time, and off-target effects of naked miRNA-based agents. To overcome these hurdles, miRNA-conjugated nanoparticles have been developed to shield the loaded agent from the surrounding environment, reduce the miRNA enzymatic inactivation or degradation, and prolong the circulation time and targeted biodistribution.^15^ Nevertheless, because of their broad biological effects, miRNA-based therapies still require extensive preclinical validation to mitigate potential safety concerns.^16^

MiRNA-26a is a member of the miRNA-26 family, which also includes miRNA-26b. It is a functional miRNA involved in the growth, development, and differentiation of several tissues.^17^ It functions as a tumor suppressor in various human malignancies, such as bladder, gastric, prostate, breast, pancreatic, and liver cancers, by targeting specific downstream genes.^18^, ^19^, ^20^, ^21^, ^22^, ^23^ It influences several processes that promote the establishment of tumor phenotypes, such as cell proliferation, senescence, migration, and metastasis.^24^

Prior research has demonstrated multiple modes of miRNA-26a delivery *in vivo* that could suppress the growth of malignant cells, including nanosystem carriers that are synthesized using single-stranded variable fragment-modified exosomes derived from human cord blood mesenchymal stem cells. These exosomes were then loaded with miRNA-26a mimics by electroporation.^25^ A different study has reported that systemic administration of miRNA-26a by an adeno-associated virus (AAV)-based delivery system in a mouse model of HCC results in inhibition of malignant cell proliferation, induction of tumor-specific apoptosis, and prevention of disease progression, all without toxicity.^23^ Accordingly, this study was conducted to evaluate the therapeutic effectiveness of miRNA-26a in the treatment of HCC utilizing nanoparticles encapsulated with the investigated miRNA in an animal model.

## 2 Materials and methods

### 2.1 Ethics approval

The use of animals adhered to the Animal Research: Reporting of *In Vivo* Experiments (ARRIVE) guidelines and the U.S. National Institutes of Health’s Guide for the Care and Use of Laboratory Animals (NIH publication No. 8523, updated 2011). All protocols were ethically reviewed and approved by the Theodor Bilharz Research Institute (TBRI) Research Ethics Committee (No. FWA 0010609; PT 541). All efforts were made to treat the mice with compassion and to follow ethical standards.

### 2.2 Materials

Sorbitan monooleate (Span® 80, SP; Sigma-Aldrich; Cat. No. 8.40123; St. Louis, MO, USA), oleylamine (OA) (Sigma-Aldrich; Cat. No. O7805; St. Louis, MO, USA), and hyaluronic acid (HA) (Merck Millipore, Cat. No. 935166; St. Louis, MO, USA) were used for nanoparticle preparation. N-Nitrosodiethylamine (ISOPAC®; Sigma-Aldrich; Cat. No. N0258; St. Louis, MO, USA) was provided at a concentration of 0.95 g/mL in a 100 mL serum bottle. For this study, it was diluted in 100 mL of saline, and the injection volume was adjusted to achieve the required dose (50 mg/kg/week). The stock solution was stored in a light-protected environment at room temperature. Isoflurane (Forane®, Baxter, UK) was used as an inhalation anesthetic to anesthetize the mice with an induction dose of 3%–5% in oxygen and a maintenance dose of 1%–3% in oxygen. Anti-cyclin D1 antibodies (Rabbit anti-human monoclonal antibodies, clone EP12, ready to use, Dako, Agilent, California, USA), HepPar 1 antibodies (Mouse anti-human monoclonal antibodies, clone OCH1E5, diluted 1:200, DakoCytomation, Carpinteria, CA, USA), arginase 1 antibodies (Mouse anti-human monoclonal antibodies, clone BBP1013, diluted 1:200, Biospes Co., Ltd., Chongqing, China), and Ki67 antibodies (Mouse anti-human monoclonal antibodies, clone MIB-1, ready to use, Dako Denmark A/S, Glostrup, Denmark) were used in the immunohistochemistry examination.

### 2.3 Nanocarriers preparation and miRNA-26a encapsulation

The preparation, conjugation, and characterization of the nanoparticles were done according to Marquez J *et al* (2018).^26^

### 2.4 Cytotoxic effects of miRNA-26a and nanoparticles

The HepG2 cell line (ATCC HB-8065; American Type Culture Collection (ATCC); USA) was cultured in 25 cm^2^ culture flasks using DMEM media supplemented with 10% fetal bovine serum (FBS). Serial dilutions of miRNA-26a, miRNA-26a-encapsulated nanoparticles, and non-encapsulated nanoparticles were added, followed by a 24-h incubation period. The cytotoxic effects were evaluated by the 3-(4,5-dimethylthiazol-2-yl)-2,5-diphenyltetrazolium bromide (MTT) assay. The experiment was conducted in quadruplicate. Dose-response curves were generated to calculate IC50 values, representing the concentration required to reduce cell viability to 50% of the control level.

### 2.5 Experimental design

A total of 80 male BALB/c strain mice, bred in-house at the animal facility of the Theodor Bilharz Research Institute (TBRI, Giza, Egypt), were used in this study. The facility operates under the oversight of the TBRI Research Ethics Committee and complies with the *Guide for the Care and Use of Laboratory Animals* and applicable national regulatory standards.

They were eight weeks old and weighed 22 ± 2 g. The mice were provided with water and a standard diet composed of 14% protein, 2% fat, and 15% fiber. The animals were kept on a 12-h light-dark cycle. The mice were acclimatized for one week prior to the initiation of DEN injection.

**Fig. 1:**
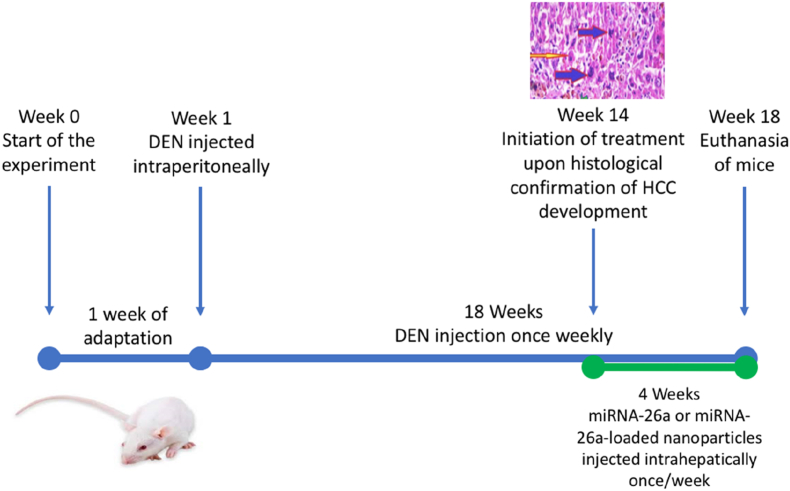
**Experimental design and timeline ofHCCinduction and treatment in BALB/c mice.** Abbreviations: DEN, diethylnitrosamine; HCC, hepatocellular carcinoma; miRNA, microRNA.

Mice enrolled in the study were randomly assigned into four groups (20 mice each, the sample size was statistically calculated to ensure adequate power for detecting significant differences between groups while adhering to the principle of using the minimum number of animals necessary) comprising: (1) a negative control group that received saline injections, (2) an HCC group that was injected intraperitoneally with diethylnitrosamine (DEN) at a dose of 50 mg/kg/week for 18 weeks; N-nitrosodiethylamine ISOPAC® (Sigma-Aldrich) was provided at a concentration of 0.95 g/mL in a 100 mL serum bottle and was diluted in 100 mL of saline, with the injection volume adjusted to attain the required dose to induce HCC. The successful establishment of the HCC model was verified based on altered liver morphology (surface irregularities and/or enlargement), and histopathological confirmation of hepatic lesions consistent with HCC, (3) miRNA-26a-treated HCC group that was injected intrahepatically with 100 nmol miRNA-26a once weekly for four weeks starting from the 14^th^ week of DEN injections, upon histological confirmation of HCC development, and (4) miRNA-26a-loaded nanoparticles-treated HCC group that was injected intrahepatically with miRNA-26a-conjugated nanoparticles once weekly for four weeks starting from the 14^th^ week of DEN injections. At the 18^th^ week of DEN injections, the mice were euthanized under mild anesthesia *via* inhalation of isoflurane, and their sera and hepatic specimens were collected (Fig. 1).

### 2.6 Evaluation of the overall health and well-being of the animals

The body weight of mice was measured before and after the experiment to determine the animals’ weight gain or loss. Body weight gain percent (BWG%) was calculated using the following formula: BWG%= (Final body weight – Initial body weight)/Initial body weight × 100.^27^

### 2.7 Biochemical analyses

Blood samples were collected from the retro-orbital plexus at euthanasia and allowed to clot at room temperature for 30 min. Samples were then centrifuged at 400 × *g* for 10 min at 4 °C, and the supernatant serum was carefully separated. The obtained serum was stored at −80 °C until further biochemical analysis.

Using commercial kits (Biodiagnostics, Egypt), alanine aminotransferase (ALT), aspartate aminotransferase (AST), alkaline phosphatase (ALP), gamma-glutamyl transferase (GGT), total bilirubin, and albumin were measured in the sera of mice according to the manufacturer’s protocol.

The levels of serological alpha-fetoprotein (AFP), des-gamma-carboxy prothrombin (DCP), vascular endothelial growth factor (VEGF), and tumor necrosis factor-α (TNF-α) were quantified in duplicates using the enzyme-linked immunosorbent assay (ELISA) kits (MyBioSource, San Diego, CA, USA), following the manufacturer’s guidelines.

### 2.8 miRNA-26a and gene expression analyses

Murine liver tissues from the examined groups were homogenized, and RNA was extracted using the QIAamp RNA Blood Mini Kit (Qiagen, Germantown, MD, USA). The purity and concentration of the RNA were evaluated using the NanoDrop™ 2000 Spectrophotometer (Thermo Fisher Scientific, Waltham, MA, USA). The A260/280 ratios of the isolated RNA ranged from 1.8 to 2.0.

The isolated RNA (1 μg) was converted into complementary DNA (cDNA) using the RevertAid First Strand cDNA Synthesis Kit (Thermo Fisher Scientific, Waltham, MA, USA) according to the manufacturer’s instructions. All quantitative polymerase chain reaction (qPCR) samples were run in triplicate.

The relative quantification value of miRNA-26a was assessed using the Taqman™ Universal Master Mix II, no UNG (Thermo Fisher Scientific, Waltham, MA, USA) based on the manufacturer’s protocol, with TaqMan® RNU6B assay for data normalization.

The expression of 70 kDa ribosomal protein S6 kinase (p70S6K), transforming growth factor beta (TGF-β), DNA methyltransferase 3 beta (DNMT3b), and Caspase-3 genes was analyzed utilizing the TaqMan gene expression master mix (Thermo Fisher Scientific, Waltham, MA, USA) in accordance with the manufacturer’s instructions. The β-actin gene served as the housekeeping gene. The primers of the examined miRNA and genes were predesigned assays obtained from Thermo Fisher Scientific (Waltham, MA, USA; Catalog No. 4427975 and 4331182, respectively).

The PCR reactions were conducted on a quantitative real-time PCR (RT-qPCR; StepOnePlus™ Applied Biosystems, Foster City, CA, USA). The comparative cycle threshold (CT) 2^−ΔΔCT^ approach was employed to calculate the relative quantification of the miRNA and genes.

### 2.9 Histopathological examination

The liver sections from the examined groups were preserved in 10% neutral buffered formalin at a ratio of 1:10 (tissue to fixative, v/v) for 24 h, then rinsed in water for 2 h, dehydrated through graded alcohol series (70%, 80%, 90%, 95%, and 100%), and cleared in xylene. The impregnation process involved immersing the specimens in pure soft paraffin at 55 °C for 2 h, followed by embedding in solid paraffin blocks. Sections (5 μm thickness) were obtained using a microtome and stained with hematoxylin and eosin.^28^

Two pathologists independently evaluated the hepatic tissue sections in a blinded manner, with one serving as a screener and the other as a consultant. The liver sections were analyzed using a light microscope (Axio scope. A1, Zeiss, Oberkochen, Germany), and photomicrographs were captured *via* a microscope camera (AxioCam, MRc5, Zeiss, Oberkochen, Germany).

### 2.10 Immunohistochemical examination

The immunohistochemical staining technique was performed utilizing the two-step method. The hepatic sections underwent deparaffinization and rehydration initially, and then the slides were subjected to antigen retrieval by heating them in pretreatment (PT) link (DAKO, Denmark A/S, Glostrup, Denmark). The antibodies investigated in this study — anti-cyclin D1, HepPar 1, arginase 1, and Ki67— were diluted according to the instructions provided by the manufacturers. The detection kit utilized was Envision Flex, manufactured by DAKO, Denmark A/S, Glostrup, Denmark. The resulting product was visualized using diaminobenzidine as the chromogen and subsequently stained with hematoxylin as a counterstain. The proportion of cells exhibiting positive responses was quantified and subjected to statistical analysis.

### 2.11 Statistical analysis

The data obtained were presented as means ± standard deviation (SD). A one-way analysis of variance (ANOVA) was conducted, followed by Tukey’s multiple comparison post hoc test, to conduct the multivariate analysis. The statistical analyses were performed using SPSS version 25 for Windows (SPSS Inc., IL, Chicago, USA). Two-tailed significance levels of *P* < 0.05 were chosen to determine the statistical differences.

## 3 Results

### 3.1 Nanocarriers preparation and miRNA-26a encapsulation

The nanocarriers loaded with miRNA-26a had a size of 151.8 ± 7.6 μm and a zeta potential of 34.5 ± 0.9 mV. The characterization of the nanoparticles was described in detail previously.^26^

### 3.2 The cytotoxic effects of miRNA-26a and nanoparticles

**Fig. 2:**
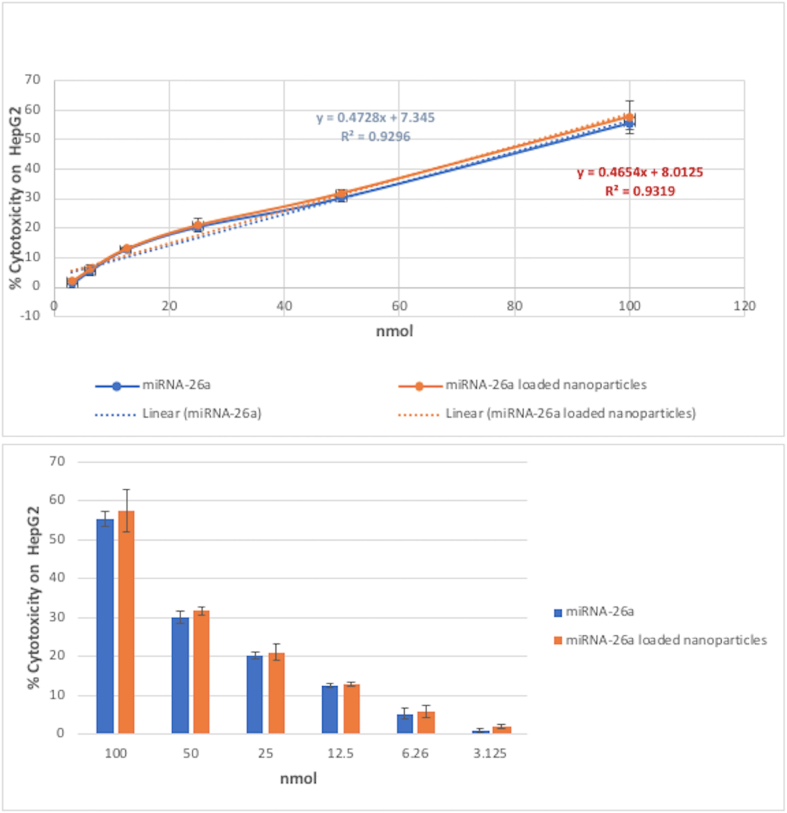
**Analysis of cytotoxicity induced by miRNA-26a and its encapsulated nanoparticles in HepG2 cells. (A)** The linear regression analysis of the % cytotoxicity of miRNA-26a and encapsulated nanoparticles on HepG2 cells. **(B)** The bar chart of the % cytotoxicity of miRNA-26a and encapsulated nanoparticles on HepG2 cells. The difference in the % cytotoxicity between miRNA-26a and encapsulated nanoparticles was statistically insignificant.

The IC50 values for miRNA-26a and encapsulated nanoparticles were 90.28 nmol and 86.82 nmol, respectively, demonstrating no significant difference in the cytotoxicity against HepG2 cells at all tested concentrations (Fig. 2). In contrast, the non-encapsulated nanoparticles exhibited no cytotoxic effect on the HepG2 cells.

### 3.3 Induction of HCC model

Mice injected with DEN developed features consistent with HCC, including nodular livers, significant elevations in serum ALT, AST, and AFP levels, and histopathological alterations confirming HCC, thereby validating the successful establishment of the model.

### 3.4 The overall health and well-being of the animals

**Fig. 3:**
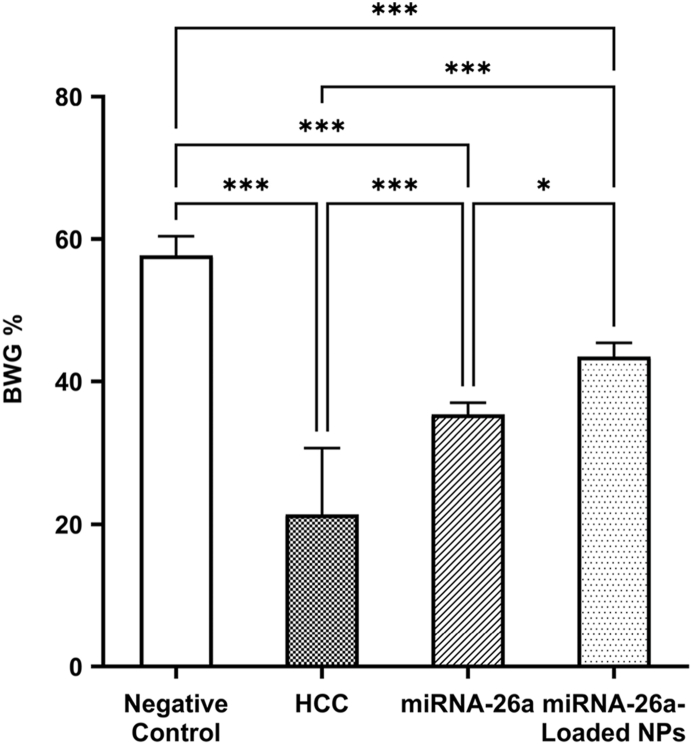
**Measurements of BWG % in the studied groups of mice.** ∗significant difference (*P* < 0.05). ∗∗∗significant difference (*P* < 0.001). Abbreviations: BWG%, body weight gain percent; HCC, hepatocellular carcinoma; NPs, nanoparticles.

It was found that the mean ± SD of BWG% significantly decreased from 57.70 ± 2.69% in the negative control group to 21.38 ± 9.30% in the HCC group (*P* < 0.001). However, treatment of HCC mice with miRNA-26a and its encapsulated nanoparticles each led to a significant increase in the BWG% to 35.37 ± 1.63% and 43.55 ± 1.92%, respectively, when compared to the HCC mice (*P* < 0.001) (Fig. 3).

### 3.5 Biochemical analyses

The DEN-induced HCC group displayed statistically significant differences in liver function tests, including increased levels of ALT, AST, ALP, GGT, and total bilirubin, along with a decrease in albumin, compared to the negative control group (*P* < 0.001). However, treatment with miRNA-26a significantly improved these parameters (*P* < 0.001) and miRNA-26a-loaded nanoparticles further enhanced these effects (*P* <0.001 for all parameters except for albumin *P* <0.01) (Table 1).

**Table 1:** Measurements of liver function tests in the studied groups of mice (*n*=20, per group).

Group	ALT (U/L)	AST (U/L)	ALP (U/L)	GGT (U/L)	Total bilirubin (mg/dL)	Albumin (g/dL)
Negative control	22.67 ± 3.50	22.67 ± 3.50	86.33 ± 6.35	29.67 ± 6.92	0.57 ± 0.08	3.75 ± 0.15
HCC	121.67 ± 9.77∗∗∗	135.17 ± 6.11∗∗∗	209.00 ± 8.97∗∗∗	126.83 ± 4.79∗∗∗	3.90 ± 0.10∗∗∗	2.58 ± 0.15^b^
miRNA-26a	85.50 ± 3.89^c^	87.50 ± 3.08^c^	152.83 ± 8.04^c^	89.33 ± 4.41^c^	2.03 ± 0.10^c^	3.20 ± 0.09^a^
miRNA-26a-loaded NPs	66.00 ± 4.65^d^	70.50 ± 3.02^d^	119.17 ± 6.91^d^	66.17 ± 3.54^d^	1.29 ± 0.10^d^	3.52 ± 0.12a, $

Data are expressed as mean ± standard deviation (SD).

Abbreviations: ALT, alanine aminotransferases; AST, aspartate aminotransferases; ALP, alkaline phosphatase; GGT, gamma-glutamyl transferase; HCC, hepatocellular carcinoma; NPs, nanoparticles.

^$^Significant increase (*P* < 0.01) *vs.* miRNA-26a-treated group.

∗∗∗Significant increase (*P* < 0.001) *vs.*negative control group*.*

a Significant increase (*P* < 0.001) *vs.* HCC group.

b Significant decrease (*P* < 0.001) *vs.* negative control group.

c Significant decrease (*P* < 0.001) *vs.* HCC group.

d Significant decrease (*P* < 0.001) *vs.* HCC and miRNA-26a-treated groups.

**Fig. 4:**
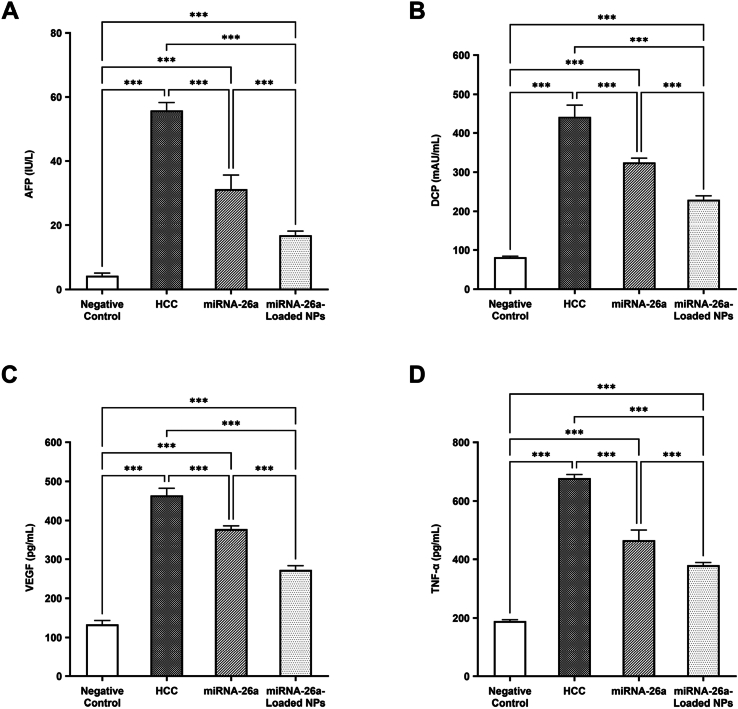
**Serum levels of cancer biomarkers in the examined groups. (A)** AFP (IU/L), **(B)** DCP (mAU/mL), **(C)** VEGF (pg/mL), and **(D)** TNF-α (pg/mL). Abbreviations: AFP, alpha-fetoprotein; DCP, des-gamma-carboxy prothrombin; VEGF, vascular endothelial growth factor; TNF, tumor necrosis factor; HCC, hepatocellular carcinoma; NPs, nanoparticles. ∗∗∗significant difference (*P* < 0.001).

The mice that received DEN injections demonstrated significant elevations in serological markers of liver cancer, angiogenesis, and proinflammatory cytokines, including AFP, DCP, VEGF, and TNF-α (All *P* < 0.001), compared to the negative control group. Administration of miRNA-26a to mice with DEN-induced HCC resulted in marked reductions in AFP, DCP, VEGF, and TNF-α levels (All *P* < 0.001). Encapsulation of nanoparticles with miRNA-26a caused a more dramatic decline in these markers in HCC mice (Fig. 4).

### 3.6 miRNA-26a and gene expression analyses

**Fig. 5:**
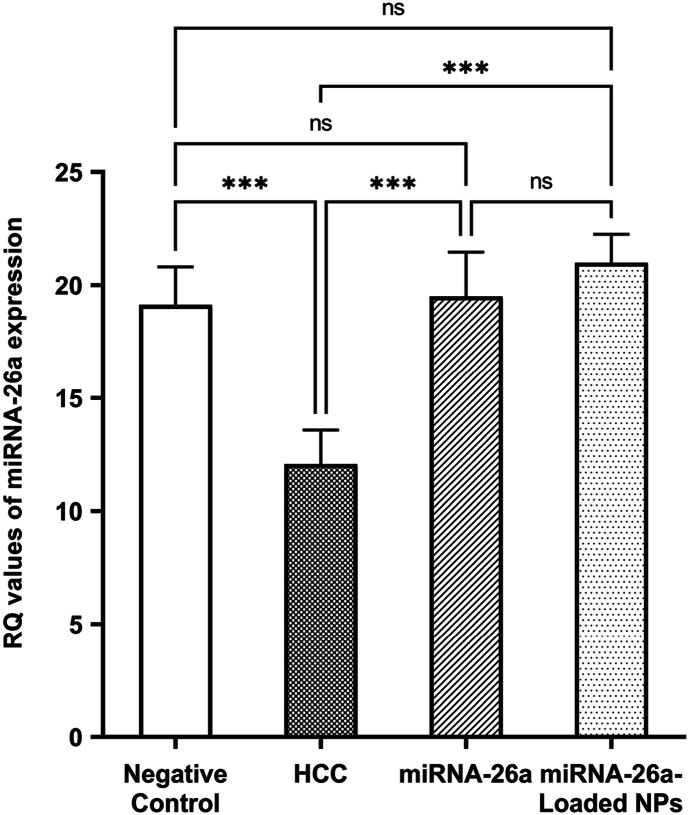
**Relative quantification (RQ) values of miRNA-26a expression in the examined groups.** Abbreviations: HCC, hepatocellular carcinoma; NPs, nanoparticles; RQ, relative quantification. ∗∗∗significant difference (*P* < 0.001); ns, not significant.

The HCC group exhibited significantly lower miRNA-26a expression compared to the negative control group (*P* < 0.001). This down-regulation was reversed by miRNA-26a injection, with further restoration of normal expression in the miRNA-26a-loaded nanoparticles-treated group (All *P* < 0.001) (Fig. 5).

**Fig. 6:**
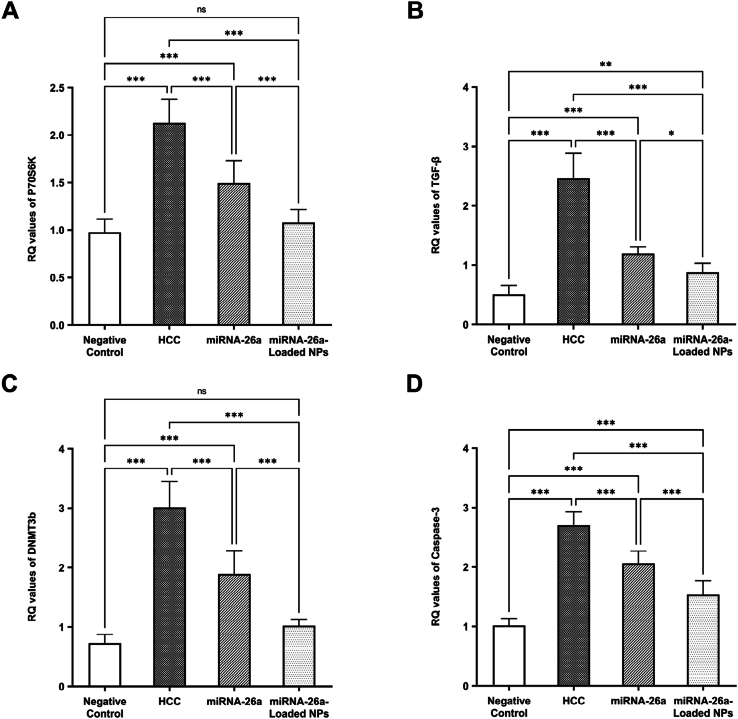
**Relative quantification (RQ) values in the examined groupsof**: **(A)***P70S6K*, **(B)***TGF-β,***(C)***DNMT3b, and***(D)***Caspase-3 genes.* Abbreviations: HCC, hepatocellular carcinoma; NPs, nanoparticles; RQ, relative quantification. ∗significant difference (*P* < 0.05). ∗∗significant difference (*P* < 0.01). ∗∗∗significant difference (*P* < 0.001). ns, not significant.

The expression of *P70S6K*, *TGF-β*, *DNMT3b, and Caspase-3* genes in the hepatic tissues was considerably greater in the HCC animal models than in the negative control group (All *P* < 0.001). The expression of these genes exhibited a notable drop in the HCC group that received miRNA-26a therapy. Furthermore, the levels of these genes were further decreased when the HCC animals were treated with nanoparticles loaded with miRNA-26a (Fig. 6).

### 3.7 Histopathological examination

**Fig. 7:**
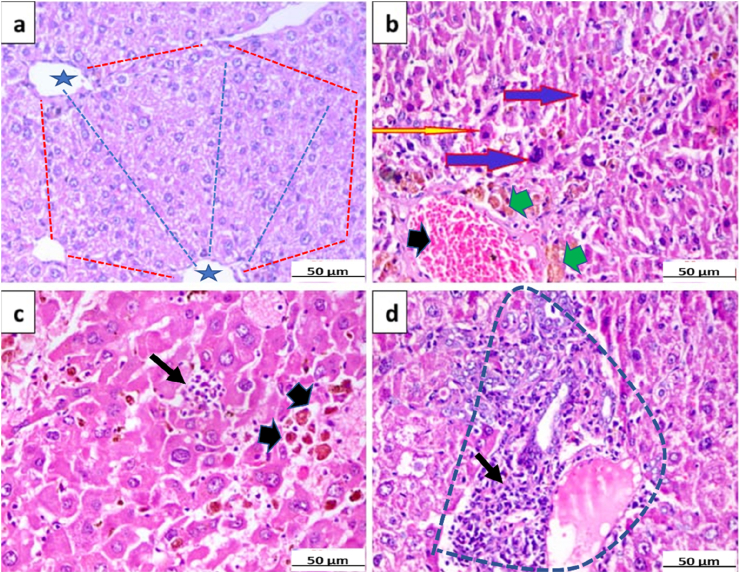
**Histopathological findings in the liver sections of the examined groups. (a)** Normal control group showed normal liver lobule hexagonal architecture (dashed red line) with normal central vein (star), radiating hepatocytes (dashed blue lines) with normal cell and nuclear size, **(b)** HCC group showing HCC with disorganized hepatic lobular architecture, cellular pleomorphism, nuclear hyperchromasia (blue arrows), apoptosis (yellow arrow), cholestasis (green arrowhead) and vascular congestion (black arrowhead), **(c)** miRNA-26a treated group showing wide areas of hepatocytic degeneration and necrosis (black thick arrow) as well as inflammatory cellular infiltration (black thin arrow), and **(d)** miRNA-26a-loaded nanoparticles treated group showing almost complete restoration of the normal hepatic architecture with mononuclear inflammatory cells (black thin arrow) infiltrating portal tracts (dashed line) (H&E stain, × 400). Abbreviations: HCC, hepatocellular carcinoma; H&E, hematoxylin and eosin.

The histopathological findings were in line with the results of the biochemical and gene expression analyses. The histopathological evaluation revealed that the livers of animals injected with DEN had constriction and significant infiltration of central veins by hepatocytes with many nuclei. Additionally, there was a disruption in the radiating hepatocyte architecture and a significant variation in the nuclear size and appearance of the liver cells compared to the normal liver. The hepatic tissues of miRNA-26a-treated mice showed marked hepatocytic necrosis, apoptosis, and infiltration by inflammatory cells with the formation of microabscesses. There were also foci of atypical bile ductular proliferation, but nuclear alterations were decreased. Whereas, the mice treated with miRNA-26a-encapsulated nanoparticles exhibited almost complete restoration of the normal hepatic architecture with mononuclear inflammatory cells infiltrating some portal tracts (Fig. 7).

### 3.8 Immunohistochemical examination

**Fig. 8:**
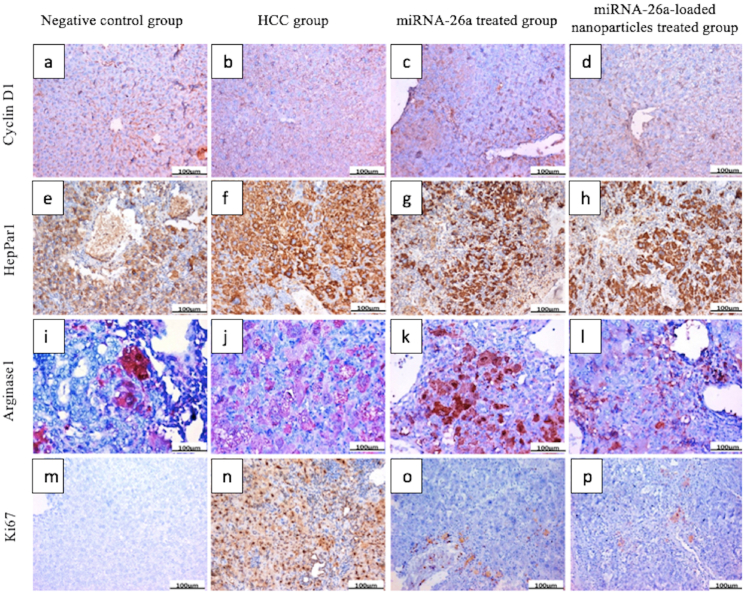
**IHC staining of liver sections. (a)** Normal control group showing moderate expression of cyclin D1 mostly in sinusoids, **(b)** HCC group showing high expression of cyclin D1 in hepatocytes, **(c)** miRNA-26a-treated group showing moderate expression of cyclin D1 in sinusoids, **(d)** miRNA-26a-loaded nanoparticles-treated group showing moderate expression of cyclin D1 in hepatocytes, **(e)** normal control group showing moderate expression of HepPar 1 in hepatocytes, **(f)** HCC group showing high expression of HepPar 1 in hepatocytes, **(g)** miRNA-26a-treated group showing moderate expression of HepPar 1 in hepatocytes, **(h)** miRNA-26a-loaded nanoparticles-treated group showing moderate expression of HepPar 1 in hepatocytes, **(i)** normal control group showing low expression of arginase 1 in some hepatocytes, **(j)** HCC group showing high expression of arginase 1 nearly in all hepatocytes, **(k)** miRNA-26a-treated group showing moderate expression of arginase 1 in hepatocytes, **(l)** miRNA-26a-loaded nanoparticles-treated group showing mild expression of arginase 1 in hepatocytes, **(m)** normal control group showing nearly negative expression of Ki67 in hepatocytes, **(n)** HCC group showing high expression of Ki67 in hepatocytes, **(o)** miRNA-26a-treated group showing low expression of Ki67 in hepatocytes, and **(p)** miRNA-26a-loaded nanoparticles-treated group showing low expression of Ki67 in hepatocytes (IHC, DAB, × 200). Abbreviations: DAB, diaminobenzidine; HCC, hepatocellular carcinoma; IHC, immunohistochemical.

The administration of DEN injections significantly upregulated the expression of cyclin D1, HepPar 1, arginase 1, and Ki67 in the majority of the mice’s hepatocytes. The expression of these proteins was substantially reduced in the group of mice with HCC that received the miRNA-26a treatment, and this reduction was even more noticeable when miRNA-26a-encapsulated nanoparticles were used to treat these mice (Fig. 8 and Table 2).

**Table 2:** The percentages of the hepatocytes’ expression of cyclin D1, HepPar 1, arginase 1, and Ki67.

Group	Cyclin D 1 (%)	HepPar 1 (%)	Arginase 1(%)	Ki67 (%)
Negative control	51.01 ± 8.24	53.73 ± 8.98	11.63 ± 4.47	1.08 ± 0.77
HCC	72.08 ± 6.66^b^	98.81 ±2.74^b^	91.13 ± 11.78^b^	68.11 ± 8.23^b^
miRNA-26a	62.22 ± 8.08^b^^,^^d^	69.17 ± 6.66^a^^,^^d^	32.60 ± 7.31^b^^,^^d^	4.11 ± 1.09^b^^,^^d^
miRNA-26a-loaded NPs	48.33 ± 7.53^c^^,^^d^	61.10 ± 9.33^c^^,^^d^	9.33 ± 3.37^c^^,^^d^	2.67 ± 2.57^d^

Abbreviations: HCC, hepatocellular carcinoma; NPs, nanoparticles.

a Significant increase (*P* < 0.05) *vs.* the negative control group.

b Significant increase (*P* < 0.01) *vs.* the negative control group.

c Significant decrease (*P* < 0.05) *vs.* the miRNA-26a-treated group.

d Significant decrease (*P* < 0.01) *vs.* the HCC group.

## 4 Discussion

In the current study, intrahepatic administration of miRNA-26a-encapsulated nanoparticles resulted in nearly total regression of liver tumors in a murine model of HCC. Additionally, cancer cell proliferation was inhibited, angiogenesis was suppressed, and tumor necrosis and apoptosis were induced. This study uncovered the therapeutic effectiveness of reinstating the disturbed expression of a miRNA in the liver. This strategy offers the advantage of miRNA being well-tolerated, as it is exclusively down-regulated in tumor cells, and hence only tumor cells are affected.

Within the context of liver cancer, miRNAs can exhibit dual roles as either tumor suppressors or oncogenes. Because a single miRNA can potentially affect numerous therapeutically relevant targets, artificially enhancing or reducing the expression level of a particular mRNA, miRNAs provide promising prospects when compared to traditional HCC therapy. One additional benefit of miRNAs is that, as they do not encode proteins, they are often not immunogenic.^29^ This type of therapy could be used in conjunction with local chemotherapy to improve the treatment efficacy. miRNA-26a stands out among these miRNAs as it can inhibit the growth of malignant cells, promote tumor-specific apoptosis, and improve the HCC sensitivity to doxorubicin.^30^

Nevertheless, due to the ability of a miRNA to influence the expression of numerous downstream targets, modifying the expression of a target miRNA may result in unfavorable off-target consequences.^31^ Therefore, it is desirable for the distribution of miRNA molecules to be both regulated and maintained, while also being selective to particular tissues. Several research groups have chosen to use non-viral methods to distribute artificially produced miRNA molecules, such as miRNA mimics or antagomirs. These molecules can be administered repeatedly locally or systemically to temporarily block the target gene expression.^32^^,^^33^ To mitigate the quick degradation of the naked molecules, they undergo modification or conjugation to enhance their stability and facilitate their targeted delivery to a specific area. They can be integrated into stable nanoparticles to guard them from disintegration, inhibit immunostimulation, and enhance endosome uptake.^34^

The purpose of miRNA replacement is to restore the level of a tumor suppressor miRNA. In the present study, miRNA-26a was forcedly expressed utilizing nanocarriers to examine its therapeutic effect on an HCC model. Similar to our findings, miRNA-26a/b restoration has been able to improve the chemosensitivity and apoptosis of HCC cell lines, which are beneficial outcomes for HCC-targeted molecular therapy.^30^ It has also been proven that the human liver cancer cell line is susceptible to the anti-proliferative effects of miRNA-26a. Additionally, it has been shown that miRNA-26a administration, *via* an adenoviral delivery system, reduces the tumorgenicity in a mouse model of HCC.^23^ Furthermore, miRNA-26 family members have been shown to inhibit carcinogenesis in c-Myc-driven B lymphoma cells and human anaplastic thyroid cancers (ATC) where temporary transfection of miRNA-26a has markedly reduced the ATC cell proliferation *in vitro*.^33^^,^^35^ miRNA-26a/miRNA-26b overexpression has suppressed cell cycle, migration, invasion, as well as glycolysis while promoting apoptosis of tongue squamous cell carcinoma (TSCC) cells *in vitro*. Additionally, it has hindered tumor progression *in vivo*.^17^ A study conducted by Gao *et al*.^36^ has revealed the inhibitory effects of miRNA-26a on the proliferation and migration of breast cancer cells. These effects were achieved by regulating the expression of MCL-1.^36^ Moreover, Deng *et al*.^37^ have discovered that miRNA-26a reduces gastric cancer metastasis and tumor growth by targeting FGF9.

On the other hand, miRNA-26a has been found to be overexpressed, maintain the proliferation, and enhance the migration in colorectal cancer (CRC) tissues, CRC-derived cell lines, and CRC animal models *via* direct regulation of PTEN.^24^ Also, its ectopic expression has promoted lung cancer cells’ migration and invasion through targeting PTEN.^38^ Additionally, some researchers have demonstrated that miRNA-26a is considerably up-regulated in glioblastoma and ovarian cancer.^39^^,^^40^

Advanced-stage tumor characteristics involve the unregulated formation of new blood vessels and the invasion of the surrounding tissues and blood vessels. In this aspect, HCC is among the most vascular solid malignant tumors, which has a strong proclivity for vascular invasion.^41^ As the tumor develops and forms solid regions, increasing the distance between tumor cells and nearby capillaries, pro-angiogenic factors are triggered, promoting the proliferation and migration of liver endothelial cells.^42^ Angiogenesis plays a crucial role in the development, progression, and metastasis of HCC and is utilized as a diagnostic criterion.^43^^,^^44^ In the present study, it was demonstrated that the induced expression of miRNA-26a was associated with the down-regulation of the angiogenesis markers, *P70S6K* gene and VEGF.

In line with previous studies, miRNA-26a regulated apoptosis and autophagy by down-regulating *TGF-β* and *Caspase-3* expression.^45^^,^^46^ Moreover, it has been demonstrated that the methyltransferases family member; *DNA methyltransferase 3b* (*DNMT3b*), which is primarily responsible for methylating specific genomic regions, is a direct target gene of miRNA-26a.^47^ Similar to our results, *DNMT3b* has been observed to be excessively expressed in many types of cancer, contributing to tumor growth and metastasis.^48^^,^^49^ Conversely, the stimulation of miRNA-26a expression has been shown to reduce *DNMT3b* expression. Dysregulation of the *DNMT3b* gene leads to complete or partial removal of methyl groups from the promoters of the *MTSS1* tumor-suppressive genes, which contributes to the development of HCC.^50^^,^^51^

Despite the promising findings of the current study, several limitations exist. Firstly, this work was conducted in a murine model, which, while beneficial for preliminary insights, may not fully reflect human liver cancer due to species-specific differences in metabolism, immune responses, and tumor microenvironments. Secondly, although nanoparticles protect miRNAs and improve their distribution, ensuring consistent and efficient delivery exclusively to liver tumor cells remains a challenge. Lastly, this study primarily demonstrates the short-term therapeutic effects of miRNA-26a on tumor development and progression. However, it lacks data on long-term outcomes, such as recurrence rates, metastasis prevention, or survival benefits. Consequently, future clinical trials are essential to validate efficacy, optimize delivery methods, and evaluate safety and off-target effects in humans.

## 5 Conclusions

The results presented herein show that the therapeutic administration of a miRNA can be used to treat existing tumors, providing a paradigm that is closely analogous to the clinical contexts in which such medicines could be employed. Therefore, it is imperative for future studies to prioritize the translation of miRNA knowledge into clinical practice for the treatment of HCC. Besides, greater emphasis should be placed on investigating the safety and reliability of miRNAs as an early therapeutic approach for HCC.

## **Authors’ contributions**

**Marwa Hassan:** Writing – review & editing, Writing – original draft, Visualization, Methodology, Formal analysis, Data curation, Conceptualization. **Inés Fernández-Piñeiro:** Writing – review & editing, Writing – original draft, Visualization, Methodology, Formal analysis, Data curation, Conceptualization. **Iker Badiola:** Writing – review & editing, Writing – original draft, Supervision, Methodology, Funding acquisition, Formal analysis, Data curation, Conceptualization. **Mohamed Elzallat:** Writing – review & editing, Writing – original draft, Visualization, Methodology, Formal analysis, Data curation, Conceptualization. **Alejandro Sánchez:** Writing – review & editing, Supervision, Methodology, Formal analysis, Data curation, Conceptualization. **Tarek Aboushousha:** Writing – review & editing, Writing – original draft, Visualization, Methodology, Formal analysis, Data curation, Conceptualization. **Ehab Hafiz:** Visualization, Methodology, Data curation. **Eman El-Ahwany:** Writing – review & editing, Writing – original draft, Supervision, Project administration, Methodology, Funding acquisition, Formal analysis, Data curation, Conceptualization.

## Data availability statement

The data that support the findings of this study are available on request from the corresponding author.

## Declaration of competing interest

The authors declare that they have no conflict of interest.
